# A critical review of the material properties guiding the clinician’s choice of root canal sealers

**DOI:** 10.1007/s00784-023-05140-w

**Published:** 2023-07-17

**Authors:** F. Cardinali, J. Camilleri

**Affiliations:** 1 Private Practice, Ancona, Italy; 2 School of Dentistry, College of Medical and Dental Sciences, University of Birmingham, Birmingham, UK

**Keywords:** Root canal sealers, Root canal obturation, Material chemistry, Hydraulic cements

## Abstract

**Objectives:**

The introduction of hydraulic cement sealers has increased the popularity of single cone obturation where the chemistry and properties of hydraulic cement sealers are crucial. This article has investigated the materials present on the market by reviewing the chemistry aiming at understanding whether these materials are optimized or have been tested appropriately.

**Methodology:**

A market search on materials called bioceramic and hydraulic sealers was undertaken. The safety data sheet and manufacturer details for every material were searched and the components were checked. The literature was searched for information about the properties of these materials based on their composition.

**Results:**

The safety data sheets and manufacturer details were imprecise with some manufacturers providing little detail on composition. From the publications reviewed, it is apparent that the materials used clinically are not optimized, and there is little evidence that the material chemistry and presentation aid the clinical technique in any way.

**Conclusions:**

There has been a rapid increase in materials identifying as bioceramics on the market. These materials have diverse chemistries, and some of the constituents are not declared. This may affect the clinical performance of these materials.

**Clinical significance:**

Smart materials developed on the clinical need which are appropriately tested are necessary for a paradigm shift in root canal obturation. It is important to use reputable materials that have been adequately researched in clinical practice.

## Introduction

The use of single cone obturation technique with hydraulic cement sealer has gained popularity as it is a simple technique and has been shown to be popular mostly among the general dental practitioners [1]. This obturation technique is sealer-based as a single gutta-percha cone is used, and the rest of the space is filled with sealer. The sealer chemistry and properties are thus important. Hydraulic cements have been advocated for use with single cone obturation [1] due to their antimicrobial characteristics [2–7]. Another feature of these sealer types is their interaction with the clinical environment they are placed in; thus, the irrigation protocol becomes of paramount importance since the irrigants used will change the dentine microstructure and also interact with the sealer modifying its properties [8–11]. There is also an interplay with the effects of solubility and leaching with the desired bioactivity and antimicrobial characteristics [12]. Although the success of root canal obturation has not been linked to the use of specific materials and techniques [13], it is clear from this systematic review [13] that the level of evidence is low, and the specific chemistry and material interactions of hydraulic cement sealers used with single cone obturation technique may be important for the success of this obturation technique. The aim of this article was to investigate which sealers are currently available for clinical use, assess their chemistry through the manufacturer instructions and safety data sheets, and evaluate how the chemistries available contributed to the required sealer properties and interaction with the clinical environment.

## Methodology

A product search of materials defined as bioceramic or hydraulic cement used as root canal sealers was conducted between January and June 2022 by FC. The search was conducted by searching for sealers that identified as bioceramics or had a hydraulic cement chemistry. The manufacturer website was searched for the composition, and details of the safety date sheets were also assessed. When this information was not available online, the manufacturer instructions supplied with the material were searched. For each material, the presentation and the constituents were listed. The components were further grouped into cement, radiopacifier, additive, vehicle, or other if the chemical could not be classified.

## Results and discussion

The main characteristics of the endodontic sealers are shown in Table 1. Throughout the search, it was clear that various hydraulic cement sealers available clinically do not have any supporting literature by the manufacturer or independent research. A number of sealers (BioSerra, Bright Endo MTA, Ceramoseal, nRoot, One Fill, ReMTA One, Sendoseal MTA, Smart Bioceramic RCS, and MTApex) were not accompanied by safety data sheets or manufacturer instructions. A number of other sealers had the composition but not the percentages listed (Bio-C Sealer, Advance RCS, Bioceramic Root Canal Sealer-SS White, and Bioceramic Root Canal Sealer-Bio-Phil). GuttaFlow Bioseal only listed zinc oxide in the safety data sheet so it was not included further since it is not considered as a hydraulic cement sealer.

**Table 1:**
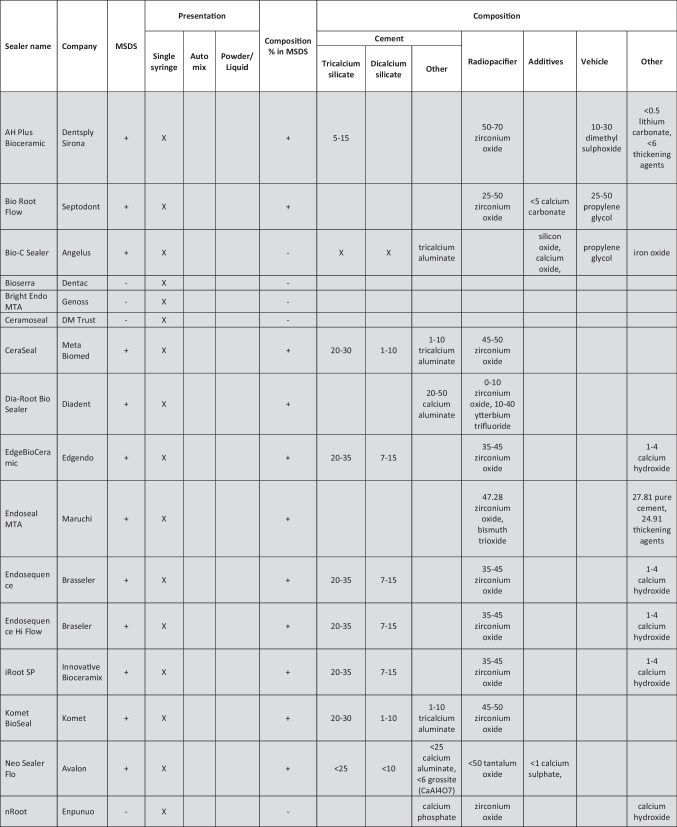

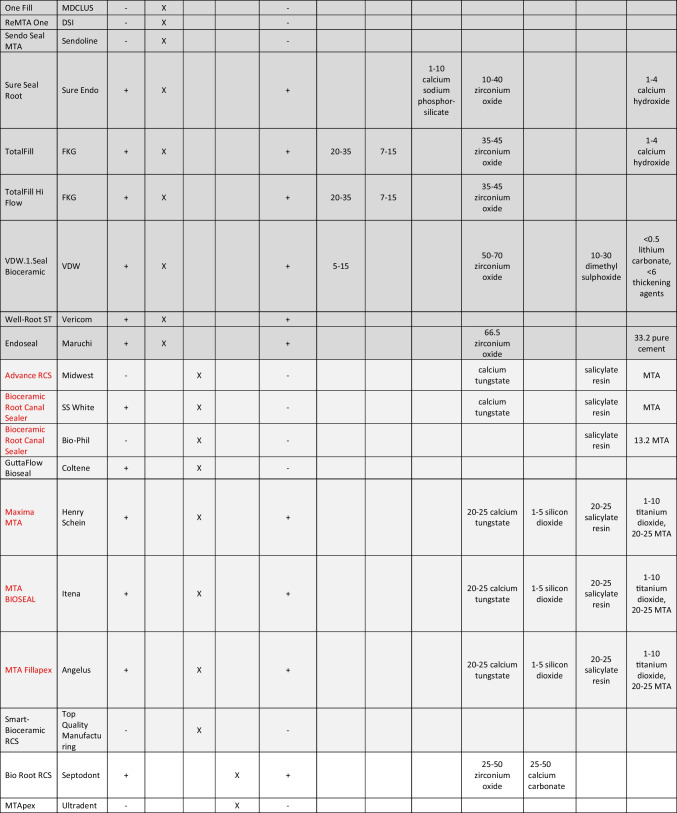
Details of different sealer types available on the market indicating the manufacturing company and the compositions declared as %. The dark grey shading is for the list of sealers presented as a single syringe, light grey for the automix syringes, and no shading for the sealers presented as a liquid and powder. The sealers containing resins are marked in red and they are not considered as hydraulic cements

### Sealer presentation

This product list was subdivided into how the sealers were presented. Most of the hydraulic cement sealers were in a single syringe format (highlighted in dark gray) and less in an automix format (highlighted in light gray). The difference between the single syringe systems and the automix is the availability of the active components. Although single syringe systems are termed pre-mixed, this is a misnomer as they require the moisture from the clinical environment to enable the setting. On the other hand, the automixed systems contain all the components required in different compartments and then are mixed and ejected through a cannula. Two manufacturers had powder/liquid formulations (shown in white background in Table 1). The delivery of the sealer and the availability of the liquid required for hydration of the cement component and setting makes the sealer types diverse and thus the single syringe systems have alternative vehicles that are water soluble to enable them to interact with the fluids in human tissues, thus allowing hydration of the materials.

### Sealer chemistry

The sealer components were subdivided into cements, radiopacifiers, additives, and vehicle as has been suggested in a recent hydraulic cement classification [14]. What could not be classified was added to other section.

#### Vehicle

The vehicle is the medium in which all the powder components are dispersed in and also defines the reaction type. All the automix systems included a resin, thus cannot be classified as hydraulic cements as their primary reaction is a resin-based reaction [14]. These have been marked in red in Table 1 and will not be considered further in this discussion. 

The vehicles of the single syringe systems were not always declared. The use of different vehicles can change the material characteristics. One such claimed case is the TotalFill and TotalFill HiFlow that are identical but have a different vehicles [15]. The manufacturer claims are that the HiFlow is heat resistant when it has been shown that both versions can be heated adequately. BioRoot and BioRoot Flow are also identical in composition except for the vehicle use as the former uses water while the latter a non-aqueous vehicle. Bio-C sealer and BioRoot Flow use propylene glycol, and VDW1.seal and AH Plus Bioceramic have dimethyl sulfoxide as a vehicle. Propylene glycol is used in toothpastes and also injectable calcium hydroxide. It is water soluble and is replaced in a clinical situation with the tissue and dentinal fluids surrounding the sealer. The main shortcoming of this system is the unreliability of the fluid transfer which may lead to delayed or non-setting. The sealer is also susceptible to interactions with the canal contents such as irrigating solutions.

The dimethyl sulfoxide is an organic solvent that is used in biological system evaluation when preparing specimens for cell culture studies. It is also used in resin systems to enhance adhesion [16–18]. Addition of dimethyl sulfoxide in proportions of > 10% to hydrophobic resin systems enhances the hydrophilicity of the material [16], thus can bond better to the wet dentine aiming at reduced long-term bond degradation. The addition of dimethyl sulfoxide in resin systems changes the physical characteristics with increased water sorption, solubility, and reduced flexural strength and elastic modulus [17]. The biocompatibility was not compromised [18]. There is no research undertaken on the use of dimethyl sulfoxide in hydraulic systems. Furthermore, the 10–30% used in AH Plus Bioceramic and VDW.1.Seal Bioceramic is much higher than the research undertaken on resin systems.

#### Radiopacifier

Radiopacifiers are added to the sealers to enhance the radiopacity and make the sealer visible on post-operative radiographs. The quality of fill which is related to material radiopacity has always been used as a measure of success of root canal therapy. Regardless of this, although root canals obturated with Resilon/Epiphany system exhibited optimal radiopacity, the treatment nonetheless failed [19, 20], indicating that the structural integrity and antimicrobial characteristics of root canal filling materials are more important than the radiopacity. The sealers contained a range of radiopacifiers, namely, zirconium oxide, tantalum oxide, ytterbium trifluoride bismuth oxide, and calcium tungstate in varying amounts even exceeding 50% in quantity. Such large amounts of filler obviously compromise the amount of cement and additives in the sealer. The Endoseal MTA contained bismuth oxide. This has been linked with tooth discoloration in contact with endodontic irrigating solutions [21] specifically sodium hypochlorite [21, 22] and also in contact with collagen in the tooth structure [23].

#### Cement

Although hydraulic cement sealers are termed bioceramic, this term is non-specific and is only a term used by companies to aid the marketing [24]. Hydraulic is a better terminology as it identifies the interaction with water for hydration and also the need of water after setting to develop the sealer properties.

Hydraulic cements can have different chemistries [14]. The tricalcium and dicalcium silicate chemistry is the one that is most investigated in dentistry as the hydration reaction results in the formation of calcium hydroxide that has been used as an antimicrobial dressing in endodontics for over a century [25]. The calcium hydroxide raises the pH and is linked to the antimicrobial characteristics of the sealers [2–11, 26], which has been the main driving force in using these sealer types in sealer-based techniques as is the single cone obturation technique. The release of calcium has been linked with the antimicrobial activity [27].

The amounts of the tricalcium silicate in all the sealers do not exceed 35% for all formulations with some being even as low as 5%. A number of sealers also include dicalcium silicate that raises the total amount of silicate in the product. The cement component does not only contribute to the desirable antimicrobial characteristics but also to the bioactivity, solubility, and leaching [12]. These properties are linked and at times even confused [28]. The release of calcium hydroxide is leaching, which is a chemical process. Bioactivity is a term that is overused in material research. Interaction of calcium hydroxide in physiological solution results in the deposition of calcium phosphate on the material surface [29]. This is a chemical interaction that is mistaken for bioactivity [30]. In fact, for hydraulic cements, the deposits on the material surface have been shown to be carbonates [31] and the deposition changes the material interaction with the substrate as the depositions on the material surface result in a reduction in pH and the relative antimicrobial characteristics as shown with MTA [32].

The solubility is the loss of particulate matter. This has been shown to be high for hydraulic cement sealers [33–36] but the testing is limited by the use of water suggested by the ISO 6876 norm [37] as the chemical changes on the surface in contact with tissue fluids may compensate for the perceived solubility of hydraulic cement sealers [36]. Depending on the environment that the material is placed in, the solubility and bioactivity can interchangeably play a role on the material resulting in a net loss or gain.

The dicalcium silicate which as indicated in Table 1 can be present in about 5–15% in a number of materials reacts with water in a similar way to the tricalcium silicate. But while tricalcium silicate is responsible for the initial hydration process, dicalcium silicate aids with the later hydration and is responsible for later material strength. The presence of tricalcium and dicalcium silicate has led to most of the manufacturers promoting the bioactivity of their products. Although there is a lack of evidence about the relation between bioactivity and amount of tricalcium and dicalcium silicate, it is evident that there is a big difference in their percentages from product to product. The quantity present effects both the bioactivity and solubility. Due to this, a further classification of the hydraulic cements in dentistry would seem necessary. The highest percentages of tricalcium silicate and dicalcium silicate declared by the manufacturer in the MSDS can be added, and the hydraulic cements can be classified as follows.

- Low charged < 20% (the sum of the highest percentages in the MSDS of tricalcium silicate and dicalcium silicate is < 20%)
- Medium charged >20%  and < 40% (the sum of the highest percentages in the MSDS of tricalcium silicate and dicalcium silicate is between 20 and 40%)
- High charged > or  = 40% (the sum of the highest percentages in the MSDS of tricalcium silicate and dicalcium silicate is > or = 40%)

Some sealers also exhibit other cementitious phases as indicated in Table 1. This includes calcium phosphate and tricalcium aluminate with varying amounts. The addition of calcium phosphate has been shown to affect the reaction of tricalcium silicate hydration, and the effects are dependent on the calcium phosphate chemistry [38]. Calcium ion release reduction has been demonstrated, which could affect the biological characteristics of the sealer [38]. The calcium aluminate can be present in small amounts of > 5% as is normally found in industrial Portland cement. Bio-C Sealer is one such material. This contributes to the hydration reaction in combination with the calcium sulfate forming ettringite and a monosulfate phase. Its presence is always found in cements that are derived from industrial Portland cement [39]. Some brands show a higher amount of calcium aluminate and even other aluminates such as grossite (CaAl4O7). Calcium aluminates are also hydraulic, and there have been claims that the use of these hydraulic materials enhances the acid resistance and reduces the solubility of the tricalcium silicate system [40]. These claims cannot be substantiated in a biological environment. Furthermore, the release of calcium from the aluminate phase cannot be assumed to lead to interaction with a biological system.

Dia-Root Biosealer does not include the tricalcium silicate. It is composed of tricalcium aluminate and radiopacifiers. The reaction of tricalcium aluminate with water leads to calcium aluminate hydrate in the absence of calcium sulfate. Calcium aluminate has been popular in the construction industry as it is easier to manufacture and develops strength faster than the Portland cement which is tricalcium silicate-based. The main limitation of the aluminate is its phase conversion when subjected to body temperatures and humidity. This phase change leads to a reduction in strength [41–43]. Addition of additives can improve the properties of calcium aluminates [44, 45].

Calcium aluminate can also be used in conjunction with tricalcium silicate cement [46] with and without the calcium sulfate [47–52]. Addition of both cement types results in a reduction in setting time [48, 50], and in excess of calcium sulfate, the cement also shows high strength and controlled expansion [47, 48]. The mixture of calcium aluminate and calcium silicate in excess of calcium sulfate would be the system used in NeoSealer Flo. This has been well reported in the construction materials literature, and such systems have also been developed as biomaterials [46–52]. The main problem is the biocompatibility as the reaction of the aluminate cement with the tricalcium silicate depleted the calcium hydroxide formed in the tricalcium silicate reaction, and this in turn results in a reduced biocompatibility tested using cell cultures [49, 50]. Calcium aluminates are known to be acid resistant; in fact, they are used in sewage systems in the construction industry. The interactions in a biological system such as the use as sealers have been shown to cause subsurface changes in material chemistry [52]. A change in the media also resulted in the deposition of a specific phase on the materials surface [53]. The cementitious interactions require further investigations specifically as it cannot assumed that what happens in the system used in the construction industry will be valid and applicable in the human body particularly with root canal sealers that have a very specific environment which changes with irrigating solutions and medicaments.

The addition of another reactive component to the hydraulic calcium silicate system interferes with the formation and leaching of calcium ions and thus also effects the solubility/bioactivity interplay. In this context, the surface interactions which the manufacturers are claiming to be bioactivity [46, 47, 49, 50].

#### Additives

The additives are used to enhance the sealer properties. Cement mixtures do not possess the necessary characteristics to be adequate sealers. The additives listed in Table 1 in the list of additives are only the ones that are known to modify the material chemistry and enhance physical characteristics. These include calcium carbonate, silicon oxide, calcium oxide, and calcium sulfate. The role of calcium sulfate has been discussed with the cement characterization. It is present as part of the Portland cement, so it is not an additive per se but an integral part of the cement as Portland cement manufacture involves the addition of calcium sulfate controls the flash setting of the tricalcium aluminate phase in the cement. In systems containing a mixture of silicates and aluminate, it is added as a reaction modifier and this makes the resultant formulation fast setting and expansive [47, 48].

The calcium oxide present in Bio-C Sealer is also not an additive but a remnant of the clinkering of Portland cement caused by improper burning. It leads to a rise in heat of hydration early in the hydration process, and this results in early release of calcium in solution [54]. The early release of calcium in solution has clinical implications. The calcium in solution is required for antimicrobial activity and also for its mineralizing potential.

Calcium carbonate is present in the BioRoot formulations. Calcium carbonate presence has never been shown for the sealer, but it has been verified for the Biodentine [54]. The calcium carbonate acts as a nucleating agent, and it also allows the release of calcium ions in solution. The hydrating cement uses the calcium carbonate particles as a nucleus and deposit around it leading to an ordered microstructure which results in enhanced physical properties [55].

Since the additives modify the cement hydration and the availability of the calcium hydroxide, the resultant material physical, chemical, and biological characteristics will change accordingly. The interaction with the substrate depends on the leaching, solubility, and chemical interactions on the material surface which is directly dependent on the formation of calcium hydroxide [27, 56].

#### Other components

Some other chemicals have been added to the materials safety data sheets. These include lithium carbonate, iron oxide, calcium hydroxide, titanium dioxide, thickening agents, pure cement, and MTA. The iron oxide is added to enhance the color in some formulations. Some Portland cements may have iron oxide as part of the clinkering process as this is part of the raw materials used to make the cement. It is lower in quantity in white cements. The iron oxide in this case is used a fluxing agent to reduce the burning temperatures during clinkering.

Calcium hydroxide is a product of hydration of the tricalcium and dicalcium silicate reaction with water. Its addition to the sealers does not seem to have a reason unless the manufacturers are either accounting for the prehydration, which occurs when the tricalcium and dicalcium silicate are in contact with the air, or else the calcium hydroxide is added as a source of calcium which is readily available in the early ages of hydration. This will have the same clinical implications as the calcium oxide and calcium carbonate additives. The use of lithium carbonate in the AH Plus Bioceramic and VDW.1.Seal Bioceramic sealers is a bit obscure. The lithium carbonate is used in management of bipolar disorders; but in industry, it is used as a ceramic glaze as it forms fluxes with silica. Cement mixtures used for tiling use lithium carbonate to accelerate the setting of the cement. Titanium dioxide is used as a whitening pigment and also as a thickener.

It is of interest to see in the material safety data sheets, the inclusion of pure cement and MTA as a chemical. MTA is a mixture of bismuth oxide and Portland cement [57, 58]. Pure cement can have any chemistry. The material safety data sheets need to be precise and declare anything that is present in substantial amounts. The same goes for thickening agents which in Endoseal MTA are present in 24.91% which is a substantial amount, and the composition is not declared.

### Sealer interaction with clinical environment

Since the hydraulic cement sealers interact with the clinical environment, the irrigation technique utilized, and the final irrigating solution is important as this determines the sealer properties after obturation. Some research has been conducted on the effect of the final irrigating solution and has shown that the solution needs to be tailor made to the sealer used. Single syringe sealers require the smear layer removal as they require the fluid from the dentin to hydrate [11]. Powder to liquid formulations such as BioRoot interact with the dentine and exert an antimicrobial effect independent of the smear layer [11]. The interaction of the hydraulic sealers with dentine seems to be mineral exchange at the interface [59, 60]. The use of phosphate-buffered saline compromises the antimicrobial effect of the sealers due to the interaction of the phosphate with the calcium hydroxide released [10]. The use of chlorhexidine enhances the antimicrobial effects of sealers, but it also causes changes to the physical properties of epoxy-resin-based, hydraulic cement, and also zinc oxide eugenol-based sealers [8, 9]. BioRoot RCS powder to liquid formulation was not affected by the presence of chlorhexidine, and its presence did not influence the antimicrobial properties and the sealer solubility reduced [61].

Although the hydraulic cement sealers set in the presence of moisture, blood, and tissue fluids have been shown to interfere with the material setting [62, 63] and the presence of blood also compromises the antimicrobial properties of the hydraulic cements [32], the root canal should be clean without any presence of blood at the time of obturation. Another factor to be taken into consideration is the presence of moisture and whether the root canal should be dried completely prior to obturation. Not much research has been undertaken in this regard. However, a recent outcome study [64] showed that the success rate was 90% when root canals were dried and filled with a hydraulic cement sealer. Furthermore, an in vitro study done in 100 recently extracted roots [65], the canals were completely dried and the sealer had set in all the samples. These two studies are indirect indicators on the recommendation to dry canals. These reported interactions indicate the need of a matched irrigation and obturation technique to enable enhanced sealer interactions [66].

There have been two previous reviews of the literature on hydraulic cement sealers. One comparing premixed materials to conventional ones [67] and a broader review evaluating the chemical, physical, and biological properties of hydraulic cements is used for various applications in endodontics [68]. Both reviews compare the hydraulic calcium silicate chemistry to other materials. The different chemistries are acknowledged in one of the reviews [68] but not discussed. That was the reason why this research was undertaken to bring the different chemistries and how the chemistry may affect the material characteristics to the attention of the researchers and clinicians using these materials.

## Conclusions

The properties of sealers used in sealer-based techniques are important as any change will influence the obturation. Knowledge of the sealer chemistry and properties is necessary for the clinicians using these materials. The interaction of hydraulic cements with the clinical environment requires a matched irrigation to obturation strategy. The presence of active leachable calcium rather than the presence of calcium silicate phases needs to be declared by the manufacturer as the material interactions by hydraulic cements and the resultant properties are dependent on this. To date, the research undertaken has been on hydraulic cements with a calcium silicate chemistry. The data pertaining to these materials cannot be translated to materials with a different chemistry. It is important to use reputable materials that have been adequately researched in clinical practice.

## References

[1] Guivarc'h M, Jeanneau C, Giraud T, Pommel L, About I, Azim AA, Bukiet F (2020). An international survey on the use of calcium silicate-based sealers in non-surgical endodontic treatment. Clin Oral Investig.

[2] Šimundić Munitić M, Poklepović Peričić T, Utrobičić A, Bago I, Puljak L (2019). Antimicrobial efficacy of commercially available endodontic bioceramic root canal sealers: a systematic review. PLoS One.

[3] Wang Z, Shen Y, Haapasalo M (2021). Antimicrobial and antibiofilm properties of bioceramic materials in endodontics. Materials (Basel).

[4] Bose R, Ioannidis K, Foschi F, Bakhsh A, Kelly RD, Deb S, Mannocci F, Niazi SA (2020). Antimicrobial effectiveness of calcium silicate sealers against a nutrient-stressed multispecies biofilm. J Clin Med.

[5] Bukhari S, Karabucak B (2019). The antimicrobial effect of bioceramic sealer on an 8-week matured enterococcus faecalis biofilm attached to root canal dentinal surface. J Endod.

[6] Wang Z, Shen Y, Haapasalo M (2014). Dental materials with antibiofilm properties. Dent Mater.

[7] Liu H, Li H, Zhang L, Wang Z, Qian J, Yu M, Shen Y (2022). In vitro evaluation of the antibacterial effect of four root canal sealers on dental biofilms. Clin Oral Investig.

[8] Kapralos V, Rukke HV, Ørstavik D, Koutroulis A, Camilleri J, Sunde PT (2021). Antimicrobial and physicochemical characterization of endodontic sealers after exposure to chlorhexidine digluconate. Dent Mater.

[9] Kapralos V, Valen H, Koutroulis A, Camilleri J, Ørstavik D, Sunde PT (2022). The dentine-sealer interface: modulation of antimicrobial effects by irrigation. Int Endod J.

[10] Arias-Moliz MT, Camilleri J (2016). The effect of the final irrigant on the antimicrobial activity of root canal sealers. J Dent.

[11] Zancan RF, Di Maio A, Tomson PL, Duarte MAH, Camilleri J (2021). The presence of smear layer affects the antimicrobial action of root canal sealers. Int Endod J.

[12] Aminoshariae A, Primus C, Kulild JC (2022). Tricalcium silicate cement sealers: do the potential benefits of bioactivity justify the drawbacks?. J Am Dent Assoc.

[13] Pirani C, Camilleri J (2022). Effectiveness of root canal filling materials and techniques for treatment of apical periodontitis - a systematic review. Int Endod J.

[14] Camilleri J (2020). Classification of hydraulic cements used in dentistry. Front Dent Med.

[15] Hadis M, Camilleri J (2020). Characterization of heat resistant hydraulic sealer for warm vertical obturation. Dent Mater.

[16] Salim Al-Ani AAS, ScarabelloStape TH, Mutluay M, Tjäderhane L, Tezvergil-Mutluay A (2019). Incorporation of dimethyl sulfoxide to model adhesive resins with different hydrophilicities: physico/mechanical properties. J Mech Behav Biomed Mater.

[17] Stape TH, Tezvergil-Mutluay A, Mutluay MM, Martins LR, do Prado RL, Pizi EC, Tjäderhane L (2016) Influence of dimethyl sulfoxide used as a solvent on the physical properties and long-term dentin bonding of hydrophilic resins. J Mech Behav Biomed Mater 64:220–8 10.1016/j.jmbbm.2016.07.003 27517666

[18] Salim Al-Ani AAS, Salim IA, Seseogullari-Dirihan R, Mutluay M, Tjäderhane L, Tezvergil-Mutluay A (2021). Incorporation of dimethyl sulfoxide into experimental hydrophilic and hydrophobic adhesive resins: evaluation of cytotoxic activities. Eur J Oral Sci.

[19] Barborka BJ, Woodmansey KF, Glickman GN, Schneiderman E, He J (2017). Long-term clinical outcome of teeth obturated with resilon. J Endod.

[20] Strange KA, Tawil PZ, Phillips C, Walia HD, Fouad AF (2019). Long-term outcomes of endodontic treatment performed with resilon/epiphany. J Endod.

[21] Camilleri J, Borg J, Damidot D, Salvadori E, Pilecki P, Zaslansky P, Darvell BW (2020). Colour and chemical stability of bismuth oxide in dental materials with solutions used in routine clinical practice. PLoS One.

[22] Camilleri J (2014). Color stability of white mineral trioxide aggregate in contact with hypochlorite solution. J Endod.

[23] Marciano MA, Costa RM, Camilleri J, Mondelli RF, Guimarães BM, Duarte MA (2014). Assessment of color stability of white mineral trioxide aggregate angelus and bismuth oxide in contact with tooth structure. J Endod.

[24] Camilleri J (2020) Materials for dentistry. Raising the bar. Front Dent Med. 10.3389/fdmed.2020.00007

[25] Dammaschke T (2008). The history of direct pulp capping. J Hist Dent Spring.

[26] El-Sherif SM, Sherief DI, El-Refai DA (2022). Evaluation of the pH, calcium ion release, and antibacterial effect of a premixed bioceramic endodontic sealer. Gen Dent.

[27] Koutroulis A, Kuehne SA, Cooper PR, Camilleri J (2019). The role of calcium ion release on biocompatibility and antimicrobial properties of hydraulic cements. Sci Rep.

[28] Elyassi Y, Moinzadeh AT, Kleverlaan CJ (2019). Characterization of leachates from 6 root canal sealers. J Endod.

[29] Niu LN, Jiao K, Wang TD, Zhang W, Camilleri J, Bergeron BE, Feng HL, Mao J, Chen JH, Pashley DH, Tay FR (2014). A review of the bioactivity of hydraulic calcium silicate cements. J Dent.

[30] Bohner M, Lemaitre J (2009). Can bioactivity be tested in vitro with SBF solution?. Biomaterials.

[31] Schembri Wismayer P, Lung CY, Rappa F, Cappello F, Camilleri J (2016). Assessment of the interaction of Portland cement-based materials with blood and tissue fluids using an animal model. Sci Rep.

[32] Farrugia C, Baca P, Camilleri J, Arias Moliz MT (2017). Antimicrobial activity of ProRoot MTA in contact with blood. Sci Rep.

[33] Silva EJ, Perez R, Valentim RM, Belladonna FG, De-Deus GA, Lima IC, Neves AA (2017). Dissolution, dislocation and dimensional changes of endodontic sealers after a solubility challenge: a micro-CT approach. Int Endod J.

[34] Torres FFE, Zordan-Bronzel CL, Guerreiro-Tanomaru JM, Chávez-Andrade GM, Pinto JC, Tanomaru-Filho M (2020). Effect of immersion in distilled water or phosphate-buffered saline on the solubility, volumetric change and presence of voids of new calcium silicate-based root canal sealers. Int Endod J.

[35] Mustafa R, Alshali RZ, Silikas N (2018). The effect of desiccation on water sorption, solubility and hygroscopic volumetric expansion of dentine replacement materials. Dent Mater.

[36] Kebudi Benezra M, Schembri Wismayer P, Camilleri J (2017). Influence of environment on testing of hydraulic sealers. Sci Rep.

[37] International Standards Organization (2012) ISO 6876. Dentistry - root canal sealing materials

[38] Schembri-Wismayer P, Camilleri J (2017). Why biphasic? Assessment of the effect on cell proliferation and expression. J Endod.

[39] Camilleri J (2007). Hydration mechanisms of mineral trioxide aggregate. Int Endod J.

[40] Primus C, Gutman JL, Tay FR, Fuks A (2021). Calcium silicate and calcium aluminate cements for dentistry reviewed. J Am Ceram Soc.

[41] Scrivener KL, Capmas A (2006) Calcium aluminate cements. P.C. Hewlett (Ed.), Lea’s Chem. Cem. Concr. (4th ed.), Elsevier, 713–782

[42] Pöllmann H (2012) Calcium aluminate cements - raw materials, differences, hydration and properties. Rev Mineral Geochem 1–82, 10.2138/rmg.2012.74.1

[43] Shirani S, Cuesta A, De la Torre AG, Diaz A, Trtik P, Holler M, Aranda MAG (2020) Calcium aluminate cement conversion analysed by ptychographic nanotomography. Cement and concrete research, 2020–11, Vol.137:106201

[44] Abd El-Hamid HK, Radwan MM (2019). Influence of nano-silica additions on hydration characteristics and cytotoxicity of calcium aluminate as biomaterial. Heliyon.

[45] Da Luz AP, Pandolfelli VC (2012). CaCO3 addition effect on the hydration and mechanical strength evolution of calcium aluminate cement for endodontic applications. Ceram Inter.

[46] Camilleri J, Montesin FE, Curtis RV, Pitt Ford TR (2006). Characterization of Portland cement for use as a dental restorative material. Dent Mater.

[47] Camilleri J (2008). Characterization and chemical activity of the Portland cement and two experimental cements with potential for use in dentistry. Int Endod J.

[48] Camilleri J (2008). Modification of MTA. Physical and mechanical properties. Int Endod J.

[49] Camilleri J (2008). The biocompatibility of modified experimental Portland cement with potential for use in dentistry. Int Endod J.

[50] Camilleri J, Montesin FE, Juszczyk AS, Papaioannou S, McDonald F, Curtis RV, Pitt Ford TR (2008). The constitution, physical properties and biocompatibility of modified accelerated Portland cement. Dent Mater.

[51] Camilleri J (2010). The sealing ability of modified experimental Portland cements with potential use in dentistry. Adv Cem Res.

[52] Camilleri J (2011). Characterisation of modified calcium silicate cements exposed to acidic environment. Mater Charact.

[53] Abd El-Hamid HK, Radwan MM (2022). Hydration behavior and formation of strätlingite compound (C2ASH8) in a bio-cement based on tri-calcium silicate and mono-calcium aluminate for dental applications: influence of curing medium. Bull National Res Center.

[54] Camilleri J, Sorrentino F, Damidot D (2013). Investigation of the hydration and bioactivity of radiopacified tricalcium silicate cement, Biodentine and MTA Angelus. Dent Mater.

[55] Grech L, Mallia B, Camilleri J (2013). Investigation of the physical properties of tricalcium silicate cement-based root-end filling materials. Dent Mater.

[56] Koutroulis A, Batchelor H, Kuehne SA, Cooper PR, Camilleri J (2019). Investigation of the effect of the water to powder ratio on hydraulic cement properties. Dent Mater.

[57] Torabinejad M, White JD (1993) Tooth filling material and method of use. Patent number: 5415547

[58] orabinejad M, White JD (1995) Tooth filling material and method of use. Patent number: 5769638

[59] Viapiana R, Guerreiro-Tanomaru J, Tanomaru-Filho M, Camilleri J (2014). Interface of dentine to root canal sealers. J Dent.

[60] KebudiBenezra M, SchembriWismayer P, Camilleri J (2018). Interfacial characteristics and cytocompatibility of hydraulic sealer cements. J Endod.

[61] Kapralos V, Sunde PT, Camilleri J, Morisbak E, Koutroulis A, Ørstavik D, Valen H (2022). Effect of chlorhexidine digluconate on antimicrobial activity, cell viability and physicochemical properties of three endodontic sealers. Dent Mater.

[62] Nekoofar MH, Davies TE, Stone D, Basturk FB, Dummer PM (2011). Microstructure and chemical analysis of blood-contaminated mineral trioxide aggregate. Int Endod J.

[63] Nekoofar MH, Stone DF, Dummer PM (2010). The effect of blood contamination on the compressive strength and surface microstructure of mineral trioxide aggregate. Int Endod J.

[64] Chybowski EA, Glickman GN, Patel Y, Fleury A, Solomon E, He J (2018). Clinical outcome of non-surgical root canal treatment using a single-cone technique with endosequence bioceramic sealer: a retrospective analysis. J Endod.

[65] Pontoriero DIK, Ferrari Cagidiaco E, Cardinali F, Fornara R, Amato M, Grandini S, Ferrari M (2022) Sealing ability of two bioceramic sealers used in combination with three obturation techniques. J Osseointegr 14(3)

[66] Fernandes Zancan R, Hadis M, Burgess D, Zhang ZJ, Di Maio A, Tomson P, Hungaro Duarte M, Camilleri J (2021). A matched irrigation and obturation strategy for root canal therapy. Sci Rep.

[67] Silva Almeida LH, Moraes RR, DornellesMorgental R, Geraldo PF (2017). Are premixed calcium silicate-based endodontic sealers comparable to conventional materials? A systematic review of in vitro studies. J Endod.

[68] Camilleri J, Atmeh A, Li X, Meschi N (2022). Present status and future directions: hydraulic materials for endodontic use. Int Endod J.

